# Systematic assessment of fluid responsiveness during early septic shock resuscitation: secondary analysis of the ANDROMEDA-SHOCK trial

**DOI:** 10.1186/s13054-020-2732-y

**Published:** 2020-01-23

**Authors:** Eduardo Kattan, Gustavo A. Ospina-Tascón, Jean-Louis Teboul, Ricardo Castro, Maurizio Cecconi, Giorgio Ferri, Jan Bakker, Glenn Hernández, The ANDROMEDA-SHOCK Investigators, Glenn Hernandez, Gustavo Ospina-Tascón, Lucas Petri Damiani, Elisa Estenssoro, Arnaldo Dubin, Javier Hurtado, Gilberto Friedman, Ricardo Castro, Leyla Alegría, Jean-Louis Teboul, Maurizio Cecconi, Giorgio Ferri, Manuel Jibaja, Ronald Pairumani, Paula Fernández, Diego Barahona, Alexandre Biasi Cavalcanti, Jan Bakker, Glenn Hernandez, Leyla Alegría, Giorgio Ferri, Nicolás Rodriguez, Patricia Holger, Natalia Soto, Mario Pozo, Jan Bakker, Deborah Cook, Jean-Louis Vincent, Bryan P. Kavanagh, Phil Dellinger, Wim Rietdijk

**Affiliations:** 1 Departmento de Medicina Intensiva, Facultad de Medicina, Pontificia Universidad Católica de Chile, Avenida Diagonal Paraguay 362, Santiago, Chile; 2 Department of Intensive Care Medicine, Fundación Valle del Lili, Universidad ICESI, Cali, Colombia; 3 Service de réanimation médicale, Hopital Bicetre, Hopitaux Universitaires Paris-Sud; Assistance Publique Hôpitaux de Paris, Université Paris-Sud, Paris, France; 4 Department of Biomedical Sciences, Humanitas Clinical and Research Center, Humanitas University, Milan, Italy; 5 Unidad de Cuidados Intensivos, Hospital Barros Luco Trudeau, Santiago, Chile; 6 Department of Intensive Care Adults, Erasmus MC University Medical Center, Rotterdam, Netherlands; 7 Department of Pulmonary and Critical Care, New York University, New York, USA; 8 Division of Pulmonary, Allergy, and Critical Care Medicine, Columbia University Medical Center, New York, USA

**Keywords:** Septic shock, Fluid responsiveness, Fluid overload, Early resuscitation

## Abstract

**Background:**

Fluid boluses are administered to septic shock patients with the purpose of increasing cardiac output as a means to restore tissue perfusion. Unfortunately, fluid therapy has a narrow therapeutic index, and therefore, several approaches to increase safety have been proposed. Fluid responsiveness (FR) assessment might predict which patients will effectively increase cardiac output after a fluid bolus (FR+), thus preventing potentially harmful fluid administration in non-fluid responsive (FR−) patients. However, there are scarce data on the impact of assessing FR on major outcomes.

The recent ANDROMEDA-SHOCK trial included systematic per-protocol assessment of FR. We performed a post hoc analysis of the study dataset with the aim of exploring the relationship between FR status at baseline, attainment of specific targets, and clinically relevant outcomes.

**Methods:**

ANDROMEDA-SHOCK compared the effect of peripheral perfusion- vs. lactate-targeted resuscitation on 28-day mortality. FR was assessed before each fluid bolus and periodically thereafter. FR+ and FR− subgroups, independent of the original randomization, were compared for fluid administration, achievement of resuscitation targets, vasoactive agents use, and major outcomes such as organ dysfunction and support, length of stay, and 28-day mortality.

**Results:**

FR could be determined in 348 patients at baseline. Two hundred and forty-two patients (70%) were categorized as fluid responders. Both groups achieved comparable successful resuscitation targets, although non-fluid responders received less resuscitation fluids (0 [0–500] vs. 1500 [1000–2500] mL; *p* 0.0001), exhibited less positive fluid balances, but received more vasopressor testing. No difference in clinically relevant outcomes between FR+ and FR− patients was found, including 24-h SOFA score (9 [5–12] vs. 8 [5–11], *p* = 0.4), need for MV (78% vs. 72%, *p* = 0.16), need for RRT (18% vs. 21%, *p* = 0.7), ICU-LOS (6 [3–11] vs. 6 [3–16] days, *p* = 0.2), and 28-day mortality (40% vs. 36%, *p* = 0.5). Only thirteen patients remained fluid responsive along the intervention period.

**Conclusions:**

Systematic assessment allowed determination of fluid responsiveness status in more than 80% of patients with early septic shock. Fluid boluses could be stopped in non-fluid responsive patients without any negative impact on clinical relevant outcomes. Our results suggest that fluid resuscitation might be safely guided by FR assessment in septic shock patients.

**Trial registration:**

ClinicalTrials.gov identifier, NCT03078712. Registered retrospectively on March 13, 2017.

## Background

Fluid administration is the first line therapy to reverse sepsis-induced tissue hypoperfusion [1, 2]. For this purpose, fluids are administered either as fluid loading at the emergency department [2], or later as fluid challenges during advanced intensive care unit (ICU)-based resuscitation [3]. However, as any other drug, fluids have a narrow therapeutic index. Insufficient fluid resuscitation may lead to progressive tissue hypoperfusion and organ dysfunction [4], while excess fluids could induce detrimental fluid overload [5–8].

Fluid responsiveness (FR) is a physiologic cardiovascular condition where an increase in preload induced by a fluid bolus leads to an increase in cardiac output (CO) by more than 10–15% [9–11]. In non-fluid responsive (FR−) patients, fluid administration does not significantly increase CO and may contribute to congestion and fluid overload. The rationale to assess FR is then to try to optimize fluid resuscitation in critically ill patients by focusing fluid boluses in FR+ hypoperfused patients and by preventing harmful fluid administration in FR− patients.

Multiple tests have been described to assess FR at the bedside [12–15]. They allow to determine the position of the patient’s heart on its systolic function curve. By applying the appropriate tests, FR can be assessed in a wide variety of clinical settings [16–18]. However, despite their relative simplicity, lack of cost, and side effects, the use of FR tests has not completely permeated into routine clinical practice [19, 20]. Moreover, recent major septic shock studies did not include systematic assessment of FR as part of the research protocols [21–25]. Only a few small pilot sepsis studies have tested the impact of FR assessment on major outcomes without conclusive results [26–29]. Indeed, a major problem is that despite a relatively sound physiological background, the concept of FR has not yet demonstrated its usefulness to improve the quality or safety of fluid administration during septic shock resuscitation.

ANDROMEDA-SHOCK is the first major study that incorporated systematic per-protocol assessment of FR [30] and thus provides the opportunity to get insight into the potential clinical relevance of this monitoring. We performed a post hoc analysis of the study dataset with the aim of exploring the relationship between FR status at baseline, fluid administration, attainment of specific targets, and clinically relevant outcomes.

## Materials and methods

The complete protocol, statistical analysis, and main results of the ANDROMEDA-SHOCK trial have been previously published [30–32]. Institutional review boards at each participating center approved the study. Informed consent was obtained directly from the patients or the surrogates.

The ANDROMEDA-SHOCK trial was a prospective, multicenter, parallel-group randomized control trial conducted in 5 Latin-American countries from March 2017 to March 2018, including a total of 424 patients with septic shock. Its main objective was to evaluate the impact on 28-day mortality of a peripheral perfusion (PPTR)- vs. lactate level-targeted resuscitation (LTR) over an 8-h intervention period. Eligible patients were included within a time frame of 4 h after the diagnosis [28], and they were subjected to a sequential and stepwise resuscitation algorithm aiming to normalize capillary refill time (CRT, < 3 s) vs. to normalize arterial lactate levels (< 2 mmol/L or at least, 20% decrease every 2 h).

The goal of fluid resuscitation in the ANDROMEDA-SHOCK trial was to restore tissue perfusion as represented by CRT or lactate targets. Fluid responsiveness was assessed before each fluid bolus and periodically during the 8-h intervention period. Specific tests were selected according to the particular clinical context and local preferences [31]. A decision algorithm was proposed to guide FR assessment in complex cases (Additional file 1). Only tests with a validated cutoff for a 10–15% increase in CO after a fluid challenge were allowed [13, 14, 17, 33, 34]. A detailed description of each used test including its cutoffs is presented in Additional file 2. Obligatory CO assessment was not part of the protocol, and thus, prediction of FR status was based on previously reported cutoffs for each test.

Fluid resuscitation was indicated and focused on perfusion target achievement. As a general principle, fluid boluses were administered only to patients in a FR+ status as part of protocolized resuscitation aimed at achieving the specific allocated target.

Fluid resuscitation was avoided in patients with a demonstrated FR− status. In these patients, further resuscitation when required was performed applying non-fluid-related steps of the protocol [31]. Whatever the FR status, further fluid boluses were not administered when perfusion targets were achieved in each group.

The first resuscitation step for FR+ patients in the PPTR group was to administer a fluid bolus of 500 mL of crystalloids every 30 min until normalizing CRT. Status of FR and central venous pressure (CVP) were reassessed after each fluid bolus, and fluids were stopped before achieving the target if the patient turned FR− or if CVP increased ≥ 5 mmHg. In the LTR group, lactate was measured every 2 h and further fluid resuscitation was decided depending on target achievement. During the 2-h time intervals, 500-mL fluid boluses were repeatedly administered every 30 min, provided that the patient did not become FR− or the CVP safety limit was not reached in the meantime.

If patients did not achieve the perfusion target for whatever reason during the fluid resuscitation step, the next protocol interventions were vasopressor or inodilator tests as previously reported [30]. All interventions had predefined safety limits [31, 35], including fluid administration in patients in whom FR could not be determined.

### Data collection and statistical analysis

Data for this study were obtained from the original ANDROMEDA-SHOCK trial database. Patients were categorized according to FR status at baseline into three groups: FR+, FR−, and non-assessable. Only patients in whom FR could be determined were considered for further analysis.

The main outcome was 28-day mortality, while clinically relevant secondary outcomes were daily sequential organ failure assessment (SOFA) scores [35], need of mechanical ventilation (MV) and renal replacement therapy (RRT), MV days, and ICU and hospital length of stay (LOS), among others [30].

Demographic and clinical data, including age, comorbidities (Charlson score), severity scores, source, and hemodynamic and perfusion variables, were registered at baseline. All protocol-related procedures and monitoring were recorded during the 8-h intervention period, including repeated FR assessment, resuscitation and total fluids, and fluid balances. FR was assessed before any fluid bolus, but also at predefined intervals during the intervention period.

After discarding normal distribution, non-parametric tests were selected to determine differences between groups. Descriptive statistics are shown as median [interquartile range] or percentage (%) accordingly. Mann-Whitney *U*, Kruskal-Wallis, chi-square, Fisher’s exact, and Z-proportion tests, with Bonferroni’s post hoc correction, were used when appropriate. Data was analyzed with Minitab v17 (Minitab Inc., State College, PA) and Graphpad Prism (Graphpad Softwares, La Joya, CA) softwares. Two-tailed *p* value < 0.05 was considered statistically significant.

## Results

Fluid responsiveness was unavailable in 76 patients at baseline, and this increased to 104 at 8 h. The group of patients categorized as with unavailable FR status over time (0 to 8 h) was the sum of early deaths and patients in whom it could be not determined mainly because of technical reasons. FR could be determined in 348 of 424 patients (82%) at randomization time. Of this sample, 242 (70%) patients were categorized as fluid responders. These FR+ patients had received a pre-protocol fluid loading of 26.7 [17–40] vs. 26.8 [20–38] mL/kg (*p* = 0.8) in FR− patients. Baseline demographic and severity characteristics of groups are shown in Table 1. A description of the whole population including the group of patients in whom FR could not be determined is shown in Additional file 3.

**Table 1:** Baseline characteristics of study participants

	Fluid responders	Non-fluid responders	*p* value
*N*	242	106	
Age (years)	63 [50–74]	66 [53–75]	0.56
Sex, *N* (%)	Female, 115 (48%)	Female, 51 (48%)	0.86
Study arm, *N* (%)	LTR, 115 (48%)	LTR, 57 (54%)	0.3
	PPTR, 127 (52%)	PPTR, 49 (46%)	
APACHE score	23 [18–29]	21 [15–27]	0.09
SOFA score	10 [7–12]	9 [7–12]	0.52
Charlson index	3 [1–5]	3 [1–5]	0.4
Sepsis origin, *N* (%)	Abdominal, 92 (38%)	Abdominal, 34 (32%)	0.3
	Pulmonary, 71 (29%)	Pulmonary, 25 (24%)	
	Urinary, 53 (22%)	Urinary, 24 (23%)	
	Other, 26 (11%)	Other, 23 (21%)	
MAP (mmHg)	66 [60–75]	67 [62–78]	0.2
Pulse pressure (mmHg)	45 [35–59]	46 [33–59]	0.76
DBP (mmhg)	51 [45–59]	52 [44–59]	0.5
CVP (mmHg)	9 [5–12]	10 [7–14]	0.001
Pre-protocol fluids (mL/kg)	26.7 [17–40]	26.8 [20–38]	0.8
Norepinephrine dose (mcg/kg/min)	0.22 [0.1–0.4]	0.21 [0.12–0.4]	0.8
Arterial lactate (mmol/L)	3.8 [2.8–5.5]	3.6 [2.8–5.5]	0.4
CRT (s)	5 [4–6]	4 [3–6]	0.002
ScvO_2_ (%)	72 [63–78]	74 [65–81]	0.27
Delta pCO_2_(v-a)	7 [5–10]	7 [5–10]	0.57

Tests: Mann-Whitney or Fisher’s exact test, accordingly

*LTR* lactate level-targeted group, *PPTR* peripheral perfusion-targeted group, *APACHE II* Acute Physiology And Chronic Health Evaluation II, *SOFA* sequential organ failure assessment score, *MAP* mean arterial pressure, *DBP* diastolic blood pressure, *CVP* central venous pressure, *CRT* capillary refill time, *ScvO*_*2*_ central venous oxygen saturation, *Delta pCO*_*2*_*(v-a)* difference between central venous carbon dioxide pressure and arterial carbon dioxide pressure

Evolution of perfusion-related parameters during the intervention period for each group is presented in Additional file 4. No difference in clinically relevant outcomes between FR+ and FR− patients at baseline was observed, including 24-h SOFA score (9 [5–12] vs. 8 [5–11], *p* = 0.4), need for MV (78% vs. 72%, *p* = 0.16), MV days (4 [2–10] vs. 5 [2–14], *p* = 0.2), need for RRT (18% vs. 21%, *p* = 0.7), ICU-LOS (6 [3–11] vs. 6 [3–16] days, *p* = 0.2), hospital LOS (13 [5–39] vs. 16 [8–28] days, *p* = 0.2), and 28-day mortality (40% vs. 36%, *p* = 0.5).

**Fig. 1:**
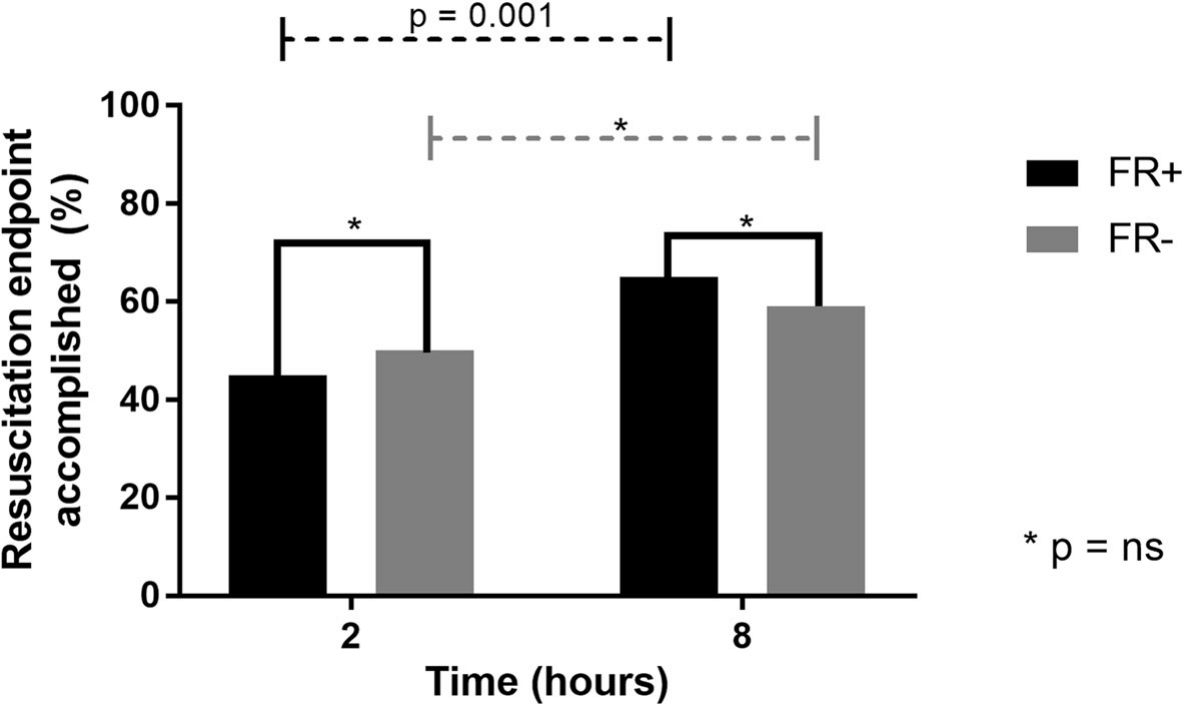
Achievement of resuscitation endpoints during the intervention period according to fluid responsiveness status at baseline. FR+, fluid responsive; FR−, non-fluid responsive

Achievement of resuscitation targets was comparable between FR+ and FR− subgroups at 2 and 8 h, but the trend was significantly higher for FR+ (Fig. 1). Use of fluids and vasoactive drugs during the study protocol is shown in Table 2. Fluid responders received significantly more fluids and completed the intervention period, with a more positive fluid balance. No statistically significant difference was found in norepinephrine requirements. On the other hand, more patients in the FR− group underwent a vasopressor or an inodilator test. Fluid balance (2056 [1012–3746] vs. 1650 [550–2560] mL; *p* = 0.02) was also significantly more positive at 24 h in the FR+ group.

**Table 2:** Resuscitation therapies during the 8-h intervention period

	Fluid responders	Non-fluid responders	*p* value
Fluids administered pre-protocol (mL)	2000 [1194–2643]	2000 [1200–2500]	0.86
FR assessments performed (*N*)	8 [7–10]	5 [5–6]	0.03
Fluid bolus 0–2 h (mL)	1000 [500–1500]	0 [0–0]	0.0001
Fluid bolus 0–8 h (mL)	1500 [1000–2500]	0 [0–500]	0.0001
Total fluids 0–8 h (mL)	2500 [1594–3840]	1748 [1090–2881]	0.003
Fluid balance 8 h (mL)	1672 [894–2842]	1244 [395–2251]	0.006
Norepinephrine dose 0 h (mcg/kg/min)	0.22 [0.1–0.4]	0.21 [0.12–0.4]	0.8
Norepinephrine dose 2 h (mcg/kg/min)	0.22 [0.09–0.45]	0.23 [0.11–0.4]	0.5
Norepinephrine dose 4 h (mcg/kg/min)	0.24 [0.1–0.42]	0.20 [0.1–0.45]	0.9
Norepinephrine dose 8 h (mcg/kg/min)	0.24 [0.1–0.45]	0.16 [0.08–0.4]	0.3
Vasopressor test (%)	74/242 (30.5%)	46/106 (43.3%)	0.02
Inodilator test (%)	33/242 (13.6%)	23/106 (21.6%)	0.08

Tests: Mann Whitney or Fisher’s exact test, accordingly

*FR* fluid responsiveness

Three hundred and twenty-eight patients were mechanically ventilated at the start of the protocol (77%). Different tests were used for the assessment of FR as depicted in Table 3. The most commonly used techniques in mechanically ventilated patients were pulse pressure variation (PPV) [36], and passive leg raising (PLR) with pulse pressure (PLR-PP) [14, 16] or velocity time integral (PLR-VTI). In non-ventilated patients, the most frequently used tests were PLR-PP and PLR-VTI. Fluid responsiveness results for each test are shown in Additional file 5.

**Table 3:** Techniques used to assess fluid responsiveness at baseline

Technique	Peripheral perfusion-targeted resuscitation (*n* = 212)	Lactate-targeted resuscitation (*n* = 212)	Total
Fluid responsiveness			
Undetermined	36 (17%)	40 (18.9%)	76 (18%)
Pulse pressure variation	73 (34.4%)	71 (33.5%)	144 (33.9%)
Passive leg rising assessed using PP, CO, or VTI	70 (33.0%)	74 (34.9%)	144 (33.9%)
End-expiratory occlusion test	3 (1.4%)	4 (1.9%)	7 (1.7%)
Inferior vena cava variation	24 (11.4%)	17 (8.0%)	41 (9.7%)
Stroke volume variation	6 (2.8%)	6 (2.8%)	12 (2.8%)

*PP* pulse pressure, *CO* cardiac output, *VTI* velocity time integral

**Fig. 2:**
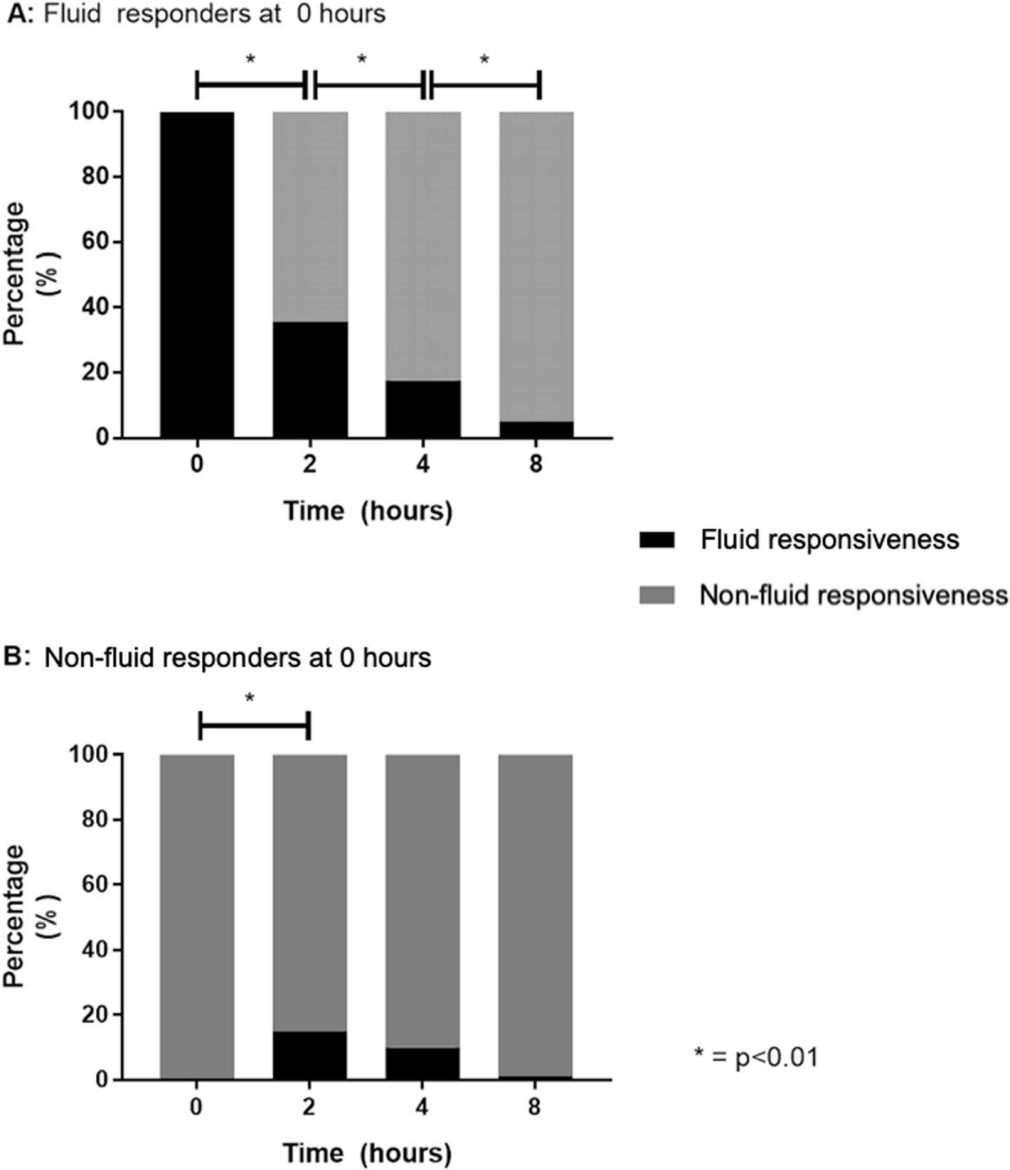
**a**, **b** Evolution of fluid responsiveness during protocolized resuscitation, according to fluid responsiveness status at baseline

Most patients evolved into a fluid-unresponsive state during the 8-h intervention period (Fig. 2). Of note, less than 15% of patients became FR+ at any time point in the FR− group, and only 13 patients that were FR+ at baseline maintained this status at the end of the intervention period.

## Discussion

Our main findings can be summarized as follows: (a) fluid responsiveness status could be determined in 82% of early septic shock patients by using diverse tests depending on the clinical context; (b) 30% of patients were already non-fluid responsive before starting ICU-based resuscitation; and (c) despite receiving less fluids, non-fluid responders at baseline resolved hypoperfusion in a similar proportion than FR+ patients by following other steps of the protocol with no difference in clinically relevant outcomes. Our results do not support a clear benefit of FR assessment on major outcomes but suggest that withholding fluid boluses in FR− patients appears to be safe.

Fluid overload has been associated with increased mortality and morbidity in patients with septic shock. Therefore, several strategies to deliver fluid resuscitation in a more physiologic, rational, and restrictive way are being tested in ongoing trials [25, 37]. Among these strategies, early use of vasopressor support [24, 38], and selecting more flow-sensitive and rapid-response targets [30], could be promising. Systematic assessment of FR might aid in preventing unnecessary fluid administration in FR− patients. However, one major concern could be the potential harm of restricting fluids during septic shock resuscitation. The 30% of patients who were FR− at baseline received around 1000 mL less fluids than FR+ patients during the first 2 h and 1500 mL less during the overall 8-h intervention period. Remarkably, they achieved resuscitation targets in the same proportion as FR+ patients, and exhibited comparable mortality and organ dysfunction improvement. Thus, these data suggest that stopping fluid resuscitation in FR− patients appears to be safe. Nevertheless, FR− patients were more frequently subjected to a protocolized vasopressor test to increase mean arterial pressure target in previously hypertensive patients, and/or to inodilators aimed at resolving hypoperfusion instead of insisting on fluid administration.

Although there is an extensive literature on the fluid responsiveness concept and background physiology, as well as on assessment techniques, the use of FR tests is not considered as a standard of care, nor has been included in recent septic shock trials. There are many possible explanations for this fact. First, some of these techniques are time-consuming, operator-dependent, not universally applicable, and with many inherent limitations. Second, there is a gray zone around the published cutoff values that somehow turns decision-making on further fluid resuscitation uncertain and complex [9]. Third, there are many misconceptions on the subject, particularly the erroneous idea that turning patients into a fluid-unresponsive state, which by definition is physiologically abnormal, is a valid objective. Therefore, the use of FR assessment could paradoxically lead to a potential fluid overload instead of preventing it. Fourth, the relevance of FR assessment for guiding therapy or the impact on major outcomes has not been demonstrated. Only 4 small randomized controlled studies including a total of 365 septic patients [26–29, 39] compared fluid responsiveness-guided resuscitation to standard techniques, and found no significant difference in major or secondary outcomes. However, the studies involved highly heterogeneous cohorts of patients and settings, and were probably underpowered to detect real differences. In this sense, although this study did not demonstrate outcome differences, it supports the idea that restricting fluid boluses in FR− septic shock patients appears at least to be safe. Future appropriately powered studies, and eventually with a randomized controlled design, should determine the definitive role of systematic FR assessment in septic shock resuscitation strategies.

The behavior of FR status during the 8-h intervention period in our cohort is intriguing. The effect of fluid boluses on CO and fluid responsiveness is thought to be transient based on previous pharmacodynamic studies [40]. This has been attributed to capillary leakage or blood redistribution from stressed to unstressed volumes [3, 9]. In our study, we did not assess CO systematically, but found that the FR+ status disappeared in almost all patients after receiving a median of only 1500 mL during the intervention period. We do not have an explanation for this finding, but the fact that only 40% of the initial FR+ patients were still fluid responsive 2 h after inclusion reinforces the idea that FR should be periodically reassessed when performing an active septic shock resuscitation [41]. On the other hand, it is unclear why so many patients exhibited a FR− state so early during resuscitation. Pre-ICU fluid loading might be responsible for this observation, but the amount of fluids received was within the limits of current recommendations. Unfortunately, the diastolic and systolic cardiac functions were not systematically evaluated whereby we cannot rule out the presence of sepsis-induced myocardial dysfunction or even previous cardiomyopathy.

This study presents several limitations. First, it has the inherent limitations of a post hoc analysis, so conclusions should be considered only as hypothesis-generating. Second, the use of some techniques might be criticized. PPV was used in one third of the patients, which is far more than expected considering the numerous limitations of this method [12]. On the other hand and despite current recommendations [42], PLR-PP was used more frequently than PLR-VTI to assess FR in spontaneous breathing patients. This could be also criticized since the changes in pulse pressure during PLR have a low sensitivity although good specificity to assess FR [9]. Indeed, a positive test (increase in PP during PLR) is reliable for detecting a FR+ state, but a negative test is not. Some centers preferred to start with PLR-PP which is much faster and easier to be applied on a 24/7 basis especially in resource-constrained settings. Anyway, the fact that the proportion of FR+ versus FR− was relatively comparable whatever the test used tends to support its use (Additional file 5). In addition, a recent retrospective study including 491 patients showed that changes in CO can be roughly predicted by increases in PP [43]. Third, our protocol did not mandate advanced hemodynamic monitoring, and therefore, data on cardiac output or stroke volume are lacking. Therefore, we acknowledge that classifying patients according to FR status might have some inherent bias since it was not confirmed by direct CO measurement or an effective fluid challenge in most of the patients. However, direct measurements of CO are not always available in clinical practice [43] and this is one of the general limitations of FR assessment techniques. Fourth, FR could not be determined in 18% of the patients, mainly because of logistic reasons. Fifth, we cannot support the external validity of our results, since only centers with experience in FR assessment were included. Sixth, we did not assess criteria of fluid overload. So, we cannot ensure that restricting fluid boluses in FR− patients prevented this complication. Despite all these limitations, it is encouraging that systematic assessment of FR was feasible in a context of mostly public hospitals in medium-income countries, and at least, this allowed to safely avoid potentially harmful fluid resuscitation in almost one third of septic shock patients.

## Conclusions

Systematic assessment allowed determination of fluid responsiveness status in more than 80% of patients with early septic shock. Fluid boluses could be stopped in non-fluid responsive patients without any negative impact on clinical relevant outcomes. Our results suggest that fluid resuscitation might be safely guided by FR assessment in septic shock patients.

## Supplementary information

**Additional file 1:** Fluid responsiveness assessment algorithm. PPV: Pulse pressure variation; PLR-VTI: Passive leg raising assessed using velocity time integral; IVCV: Inferior vena cava variation; SVCV: Superior vena cava variation; EEOT: end-expiratory occlusion test; SVV: stroke volume variation; ARDS: Acute respiratory distress syndrome; CO: Cardiac output; CI: Cardiac index; VTI: Velocity time integral; Vt: Tidal volume; PBW: Predicted body weight.

**Additional file 2:** Technical details of different fluid responsiveness assessment techniques.

**Additional file 3:** Baseline characteristics including the subgroup of patients in whom FR could not be assessed.

**Additional file 4:** Evolution of perfusion parameters during the first 24 h.

**Additional file 5:** Distribution of fluid responsiveness at baseline according to the test used.

## Data Availability

The datasets generated and/or analyzed during the current study are not publicly available until February 2020 when we expect to have published all sub-studies, but are available before from the corresponding author on reasonable request.

## Abbreviations

APACHE II: Acute Physiology And Chronic Health Evaluation II
ARDS: Acute respiratory distress syndrome
CO: Cardiac output
CRT: Capillary refill time
CVP: Central venous pressure
DBP: Diastolic blood pressure
Delta pCO2(v-a): Difference between central venous carbon dioxide pressure and arterial carbon dioxide pressure
EEOT: End-expiratory occlusion test
FR: Fluid responsiveness
FR−: Non-fluid responsive
FR+: Fluid responsive
ICU: Intensive care unit
IVCV: Inferior vena cava variation
LOS: Length of stay
MAP: Mean arterial pressure
MV: Mechanical ventilation
PLR: Passive leg raising
PLR-PP: Passive leg raising assessed using pulse pressure
PLR-VTI: Passive leg raising assessed using velocity time integral
PPV: Pulse pressure variation
RRT: Renal replacement therapy
ScvO2: Central venous oxygen saturation
SOFA: Sequential organ failure assessment
SSC: Surviving Sepsis Campaign
SVV: Stroke volume variation
