# Supplementary figures and images for: Systematic assessment of fluid responsiveness during early septic shock resuscitation: secondary analysis of the ANDROMEDA-SHOCK trial
Source: Crit Care. 2020 Jan 23;24:23. doi: 10.1186/s13054-020-2732-y (PMC6979284; doi:10.1186/s13054-020-2732-y)

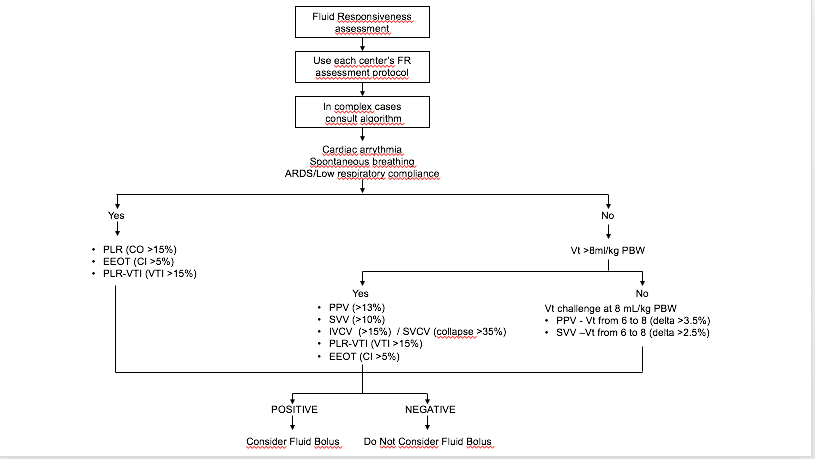


**Additional File 1**

Supplement: Supplementary file 1 — Additional file 1: Fluid responsiveness assessment algorithm. PPV: Pulse pressure variation; PLR-VTI: Passive leg raising assessed using velocity time integral; IVCV: Inferior vena cava variation; SVCV: Superior vena cava variation; EEOT: end-expiratory occlusion test; SVV: stroke volume variation; ARDS: Acute respiratory distress syndrome; CO: Cardiac output; CI: Cardiac index; VTI: Velocity time integral; Vt: Tidal volume; PBW: Predicted body weight. [file 13054_2020_2732_MOESM1_ESM.docx]
