# Supplementary material for: Systematic assessment of fluid responsiveness during early septic shock resuscitation: secondary analysis of the ANDROMEDA-SHOCK trial
Source: Crit Care. 2020 Jan 23;24:23. doi: 10.1186/s13054-020-2732-y (PMC6979284; doi:10.1186/s13054-020-2732-y)
Supplement: Supplementary file 2 — Additional file 2: Technical details of different fluid responsiveness assessment techniques. [file 13054_2020_2732_MOESM2_ESM.docx]

Additional File 2.- Technical details of different fluid responsiveness assessment techniques

| Test | Details |
| --- | --- |
| PPV | Measurable in mechanically ventilated patients, with no spontaneous breathing efforts, tidal volume equal or higher than 8 ml/kg of ideal body weight, with cardiac sinus rhythm, and no cause of low respiratory compliance. Measured through a respiratory cycle, with the formula: (PPmax-PPmin)/((PPmax+PPmin)/2). Value > 13% considered significant. If TV less than 8ml/kg, perform a tidal volume challenge, transiently elevating TV to 8 ml/kg [33,36]. |
| PLR PP | Measurable in spontaneously breathing or mechanically ventilated patients. Patient must be quickly elevated from semi-recumbent position to a reverse-trendelemburg position with legs in 45°. Changes in pulse pressure must be registered before 1 minute of position change. with the formula: (PPmax-PPmin)/((PPmax+PPmin)/2). Value > 12% considered significant. Use with caution in patients with abdominal or inferior extremity surgical interventions and in abdominal hypertension. [14] |
| PLR VTI | Measurable in spontaneously breathing or mechanically ventilated patients. Patient must be quickly changed from semi-recumbent position to a reverse-Trendelenburg position with legs in 45°. Changes in VTI must be registered before 1 minute of position change. Use the formula: (VTImax-VTImin)/ (VTImax+VTImin)/2). Value > 15% considered significant. Use with caution in patients with abdominal or inferior extremity surgical interventions and in abdominal hypertension. [14] |
| IVCV | With a transthoracic echocardiographic probe, subcostal longitudinal view of IVC is obtained. With M-Mode, maximal and minimal diameter are registered. Value are obtained with the formula (Dmax-Dmin)/Dmin). Values > 15% are considered significant during mechanical ventilation [34]. Collapsibility of IVC during spontaneous breathing has also been used satisfactorily to predict fluid responsiveness, with suggested values of >40% [17]. |
| EEOT | Measurable in mechanically ventilated patients. Output measured with a direct cardiac output monitor. A 15 seconds or higher end expiratory pause is performed (with no patient-induced effort). Value > 5% increase of CO is considered significant. [13] |
| SVV | Measurable in mechanically ventilated patients, with no spontaneous breathing efforts, tidal volume equal or higher than 8 ml/kg of ideal body weight, with cardiac sinus rhythm, and no cause of low respiratory compliance. Output measured with minimally invasive direct cardiac output monitors, which use proprietary formula of pulse-contour analysis. Value > 10% considered significant. If TV less than 8ml/kg, perform a tidal volume challenge, transiently elevating TV to 8 ml/kg. [33,36] |

PPV: Pulse pressure variation, PLR-PP: Passive leg raising with pulse pressure; PLR-VTI: Passive leg raising with velocity time integral; IVCV: Inferior vena cava variation; EEOT: end-expiratory occlusion test; SVV: stroke volume variation. CO: Cardiac Output; TV: Tidal volume
