# Supplementary material for: Systematic assessment of fluid responsiveness during early septic shock resuscitation: secondary analysis of the ANDROMEDA-SHOCK trial
Source: Crit Care. 2020 Jan 23;24:23. doi: 10.1186/s13054-020-2732-y (PMC6979284; doi:10.1186/s13054-020-2732-y)
Supplement: Supplementary file 3 — Additional file 3: Baseline characteristics including the subgroup of patients in whom FR could not be assessed. [file 13054_2020_2732_MOESM3_ESM.docx]

**Additional File 3: Baseline characteristics including the subgroup of patients in whom FR could not be assessed.**

|  | FR assessable | FR not assessable | p |
| --- | --- | --- | --- |
| N° | 348 | 76 |  |
| Age (years) | 65 [51-75] | 73 [60-82] | 0.0004 |
| Sex N (%) | Female 166 (48) | Female 33 (43) | 0.6 |
| APACHE score | 22 [17-28] | 20 [16-25] | 0.14 |
| SOFA score | 10 [7-12] | 9 [7-11] | 0.1 |
| Charlson Index | 3 [1-5] | 3 [1-5] | 0.86 |
| Sepsis origin  N (%) | Pulmonary 96 (28) | Pulmonary 32 (42) | 0.02 |
|  | Abdominal: 126 (36) | Abdominal: 23 (30) | 0.3 |
|  | Urinary: 77 (22) | Urinary: 10 (13) | 0.4 |
|  | Other: 49 (14) | Other: 11 (14) | 0.3 |
| MAP (mmHg) | 66 [60-76] | 67 [60-77] | 0.84 |
| Pulse Pressure mmHg | 45 [34-59] | 43 [33-54] | 0.6 |
| CVP (mmHg) | 9 [6-12] | 8 [6-14] | 0.9 |
| Pre-protocol fluids (mL) | 2000 [1200-2500] | 2140 [1500-3275] | 0.03 |
| Norepinephrine dose (mcg/kg/min) | 0.22 [0.1-0.4] | 0.2 [0.1-0.31] | 0.1 |
| Arterial lactate (mmol/L) | 3.6 [2.8-5.5] | 3.1 [2.5-4.9] | 0.02 |
| CRT (s) | 5 [4-6] | 4 [3-5] | 0.07 |
| ScvO_2_ (%) | 73 [64-79] | 72 [65-80] | 0.44 |
| Delta pCO_2_(v-a) | 7 [5-10] | 6 [4-9] | 0.03 |
| 28 day Mortality N (%) | 135 (39%) | 35 (46%) | 0.2 |
| ICU LOS (days) | 6 [3-12] | 6 [3-10] | 0.8 |
| Fluid Bolus 8h (mL) | 1000 [400-2000] | 1000 [0-1625] | 0.24 |
| Fluid balance 8h (mL) | 1515 [698-2629] | 1095 [191-2169] | 0.007 |

Test: Mann-Whitney U test or Fisher’s exact test, accordingly.

FR: fluid responsiveness; APACHE II: Acute Physiology And Chronic Health Evaluation II; SOFA: Sequential organ failure Assessment score; MAP: Mean arterial pressure; CVP: Central venous pressure; CRT: Capillary refill time; ScvO_2_: central venous oxygen saturation; Delta pCO_2_(v-a) difference between central venous carbon dioxide pressure and arterial carbon dioxide pressure; ICU: intensive care unit, LOS: length of stay.
