# Supplementary material for: Systematic assessment of fluid responsiveness during early septic shock resuscitation: secondary analysis of the ANDROMEDA-SHOCK trial
Source: Crit Care. 2020 Jan 23;24:23. doi: 10.1186/s13054-020-2732-y (PMC6979284; doi:10.1186/s13054-020-2732-y)
Supplement: Supplementary file 4 — Additional file 4: Evolution of perfusion parameters during the first 24 h. [file 13054_2020_2732_MOESM4_ESM.docx]

**Additional File 4: Evolution of perfusion parameters during the first 24 hours**

| Parameter | Fluid responders | Non-fluid responders | p |
| --- | --- | --- | --- |
| CRT 0h (s) | 5 [4-6] | 4 [3-6] | 0.008 |
| CRT 2h (s) | 4 [3-5] | 3 [2.5-4] | 0.02 |
| CRT 4h (s) | 3 [2-4] | 3 [2-4] | 0.2 |
| CRT 8h (s) | 3 [2-3] | 2.5 [2-4] | 0.12 |
| CRT 24h (s) | 3 [2-3] | 2[2-3] | 0.08 |
| Lactate 0h (mmol/L) | 3.8 [2.8-5.5] | 3.6 [2.8-5.5] | 0.4 |
| Lactate 2h (mmol/L) | 3.3 [2.2-5.2] | 2.6 [2.1-4.2] | 0.14 |
| Lactate 4h (mmol/L) | 3 [2-4.6] | 2.5 [1.9-3.8] | 0.1 |
| Lactate 8h (mmol/L) | 2.5 [1.8-4.4] | 2.2 [1.6-3.3] | 0.13 |
| Lactate 24h (mmol/L) | 1.8 [1.4-2.9] | 1.9 [1.4-2.5] | 0.88 |
| Delta pCO_2_(v-a) 0h | 7 [5-10] | 7 [5-10] | 0.21 |
| Delta pCO_2_(v-a) 8h | 6 [4.5-8} | 6 [4-8} | 0.28 |
| Delta pCO_2_(v-a) 24h | 5.2 [4-7] | 5.1 [4-8] | 0.94 |
| ScvO_2_ 0h (%) | 72 [63-78] | 74 [65-81] | 0.5 |
| ScvO_2_ 8h (%) | 72 [65-77] | 74 [68-80] | 0.06 |
| ScvO_2_ 24h (%) | 73 [67-79] | 76 [70-80] | 0.06 |

Test: Mann-Whitney U test.

CRT: capillary refill time; ScvO_2_: central venous oxygen saturation; Delta pCO_2_(v-a) difference between central venous carbon dioxide pressure and arterial carbon dioxide pressure.
