# Supplementary material for: Systematic assessment of fluid responsiveness during early septic shock resuscitation: secondary analysis of the ANDROMEDA-SHOCK trial
Source: Crit Care. 2020 Jan 23;24:23. doi: 10.1186/s13054-020-2732-y (PMC6979284; doi:10.1186/s13054-020-2732-y)
Supplement: Supplementary file 5 — Additional file 5: Distribution of fluid responsiveness at baseline according to the test used. [file 13054_2020_2732_MOESM5_ESM.docx]

**Additional File 5: Distribution of fluid responsiveness at baseline according to the test used.**

| Test used | FR+ | FR- |
| --- | --- | --- |
| PPV | 68% | 32% |
| PLR-PP | 79% | 21% |
| PLR-VTI | 61% | 39% |
| IVCV | 61% | 39% |
| EEOT | 88% | 12% |
| SVV | 70% | 30% |

FR+: Fluid responsive; FR-: Non-fluid responsive; PPV: Pulse pressure variation; PLR-PP: Passive leg raising assessed using pulse pressure; PLR-VTI: Passive leg raising assessed using velocity time integral; IVCV: Inferior vena cava variation; EEOT: end-expiratory occlusion test; SVV: stroke volume variation.
